# Google Bard and ChatGPT in Orthopedics: Which Is the Better Doctor in Sports Medicine and Pediatric Orthopedics? The Role of AI in Patient Education

**DOI:** 10.3390/diagnostics14121253

**Published:** 2024-06-13

**Authors:** Riccardo Giorgino, Mario Alessandri-Bonetti, Matteo Del Re, Fabio Verdoni, Giuseppe M. Peretti, Laura Mangiavini

**Affiliations:** 1 Residency Program in Orthopaedics and Traumatology, University of Milan, 20122 Milan, Italy; 2 IRCCS Ospedale Galeazzi Sant’ambrogio, 20157 Milan, Italy; matteodelre91@gmail.com (M.D.R.); verdonifabio@gmail.com (F.V.); giuseppe.peretti@unimi.it (G.M.P.); laura.mangiavini@unimi.it (L.M.); 3 Department of Plastic Surgery, University of Pittsburgh Medical Center, 1350 Locust Street, Pittsburgh, PA 15213, USA; m.alessandribonetti@gmail.com; 4 Department of Biomedical Sciences for Health, University of Milan, 20122 Milan, Italy

**Keywords:** orthopedics, sports medicine, pediatric orthopedics, diagnostics, ACL, flat foot, AI, natural language processing

## Abstract

Background: This study evaluates the potential of ChatGPT and Google Bard as educational tools for patients in orthopedics, focusing on sports medicine and pediatric orthopedics. The aim is to compare the quality of responses provided by these natural language processing (NLP) models, addressing concerns about the potential dissemination of incorrect medical information. Methods: Ten ACL- and flat foot-related questions from a Google search were presented to ChatGPT-3.5 and Google Bard. Expert orthopedic surgeons rated the responses using the Global Quality Score (GQS). The study minimized bias by clearing chat history before each question, maintaining respondent anonymity and employing statistical analysis to compare response quality. Results: ChatGPT-3.5 and Google Bard yielded good-quality responses, with average scores of 4.1 ± 0.7 and 4 ± 0.78, respectively, for sports medicine. For pediatric orthopedics, Google Bard scored 3.5 ± 1, while the average score for responses generated by ChatGPT was 3.8 ± 0.83. In both cases, no statistically significant difference was found between the platforms (*p* = 0.6787, *p* = 0.3092). Despite ChatGPT’s responses being considered more readable, both platforms showed promise for AI-driven patient education, with no reported misinformation. Conclusions: ChatGPT and Google Bard demonstrate significant potential as supplementary patient education resources in orthopedics. However, improvements are needed for increased reliability. The study underscores the evolving role of AI in orthopedics and calls for continued research to ensure a conscientious integration of AI in healthcare education.

## 1. Introduction

In the ever-evolving world of orthopedics and sports medicine, new technologies are fundamentally transforming both medical practice and patient experiences. Alongside traditional innovations in orthopedic implants and surgical techniques, we are witnessing the introduction of cutting-edge technologies such as 3D printers and augmented reality, which are revolutionizing treatment design and customization [1,2,3,4,5,6]. Among these various technologies, the Internet has become a significant source for patients, who often rely on this virtual platform to find answers to their medical inquiries [7,8]. However, one of the most significant changes is represented by the rise of artificial intelligence (AI) and chatbots that combine AI with natural language processing (NLP), such as ChatGPT (OpenAI, San Francisco, CA, USA), and Google Bard (Google LLC., Mountain View, CA, USA) [9,10,11,12].

These chatbots are increasingly becoming a vital source of medical information for patients, who often use them to obtain answers to their questions and access healthcare advice [9,10,11,12]. Particularly in the fields of sports medicine and pediatric orthopedics, where injury management and performance enhancement require a thorough understanding of musculoskeletal anatomy and injuries, access to timely and accurate information is crucial for optimizing outcomes. Among the various chatbots available, ChatGPT-3.5 and Google Bard stand out for their unique features. While ChatGPT-3.5 relies on training data up to September 2021, offering a vast knowledge base but potentially outdated information, Google Bard has the ability to access real-time data on the Internet, ensuring fresher information [10,11,12]. This difference is particularly relevant in the context of sports medicine, where new discoveries and advances in injury management can directly influence clinical decisions.

However, despite the potential of these platforms to offer knowledge comparable to that of a medical graduate, concerns arise regarding the dissemination of inaccurate information [12]. This risk is amplified in the context of pediatric orthopedics, where communication with patients’ parents is crucial, and the spread of inaccurate information could influence clinical decisions and compromise trust in the doctor–patient relationship. Therefore, while the use of chatbots as a source of medical information continues to grow, it is essential to ensure supervision and review by qualified healthcare professionals. With these premises, the current study aims to compare ChatGPT and Google Bard as potential sources of information for patients or patients’ parents, evaluating the quality of responses to frequently asked questions in the fields of sports medicine and pediatric orthopedics.

## 2. Materials and Methods

To evaluate sports medicine, we conducted a Google search using the keyword “anterior cruciate ligament” (ACL), with the search history previously cleared. The first ten questions under the “people also ask” section, were recorded. Duplicate questions with the same meaning were removed. The ten questions were then presented to ChatGPT-3.5 and Google Bard. A new user account was created for this study, and the chat history was cleared before each question to minimize potential bias from ChatGPT and Google Bard’s memory retention feature. To assess the quality of the responses, we asked two expert orthopedic surgeons in sports medicine to rate the quality of responses in their native language provided by ChatGPT and Google Bard on a scale of 1 (poor quality) to 5 (excellent quality) based on the validated Global Quality Score (GQS) [13] (Table 1).

For the field of pediatric orthopedics, we selected an extremely important topic in pediatric orthopedics, namely, flat feet, and subsequently conducted a Google search with the search history previously cleared. We then recorded the first ten questions in the “people also ask” section. All duplicate questions with the same meaning were removed. The ten questions were then posed to ChatGPT-3.5 and Google Bard. Even in this case, to eliminate any bias and minimize the potential effect of the memory-retention feature of ChatGPT and Google Bard, a new user account was created, and the chat history was cleared before each question. To assess the quality of the responses, we asked two expert orthopedic surgeons in pediatric orthopedics to evaluate the quality of responses provided by both chatbots in their native language on a scale from 1 (poor quality) to 5 (excellent quality) based on the Validated Global Quality Score (GQS) [13] (Table 1). The respondents were blinded to the sources of information, and each respondent was unaware of the other’s evaluation. In both cases, the respondents were kept anonymous with respect to the sources of information. Misinformation spread was defined as the dissemination of erroneous, misleading or false information.

### Statistical Analysis

The analysis was performed using SPSS software version 26 (IBM SPSS Statistics, Chicago, IL, USA). To determine whether there were statistically significant differences between the average scores of responses generated by Google Bard and ChatGPT, we conducted independent-sample t-tests. We set the significance level (alpha) at 0.05 for all statistical tests. A *p*-value less than 0.05 was considered indicative of a statistically significant difference between the groups.

## 3. Results

In sports medicine, the average score for responses provided by Google Bard was 4 ± 0.78, while the average score for responses generated by ChatGPT was 4.1 ± 0.7. This demonstrated that both chatbots provided good-quality answers that covered the most important topics and were useful to patients. The difference between the two platforms was not statistically significant (*p* = 0.6787). The questions for ChatGPT and Google Bard are reported in Table 2 and Table 4.

For pediatric orthopedics, the average score for responses provided by Google Bard was 3.5 ± 1, while the average score for responses generated by ChatGPT was 3.8 ± 0.83. The difference between the two platforms was not statistically significant (*p* = 0.3092). These results demonstrated that both chatbots provided moderate- to good-quality responses that covered some information, but important topics were sometimes missing. The question that received the lowest rating was undoubtedly the one related to the timing of operating on flat feet; both experts gave it a Global Quality Score of 2 to 3. The questions for ChatGPT and Google Bard are listed in Table 3. In this case, both experts agreed that ChatGPT proved to be more comprehensive and concise, with no reported instances of incorrect information. Results are better presented in Table 3 and Table 4.

## 4. Discussion

This study thoroughly examined the use of chatbots in orthopedics, with a particular focus on sports medicine and pediatric orthopedics. According to the feedback provided by two expert orthopedic surgeons in these fields, both sources demonstrate promising potential for the use of AI technology as an advanced educational tool for patients.

Regarding sports medicine, ChatGPT’s responses were found to be more readable and concise, according to the feedback from the two expert orthopedic surgeons. Neither of the respondents encountered misinformation spread, i.e., the dissemination of erroneous, misleading or false information. This is an extremely positive aspect, considering that there have been reports of AI hallucinations in which incorrect responses are convincingly described [14]. However, this phenomenon cannot be entirely ruled out, given that the training of these chatbots may potentially be based on “grey literature.” Consequently, despite the accuracy of the information provided in this study, we emphasize that only a user capable of proofreading the content of the responses can recognize any incorrect information. This is arguably the aspect to keep most under control, since incorrect information assumed to be correct would undoubtedly have a negative impact on patient education. The limitations of ChatGPT in relation to its training data are evident in its inability to provide reliable sources for the information it presents. Often, the links provided by ChatGPT are inaccessible. At the same time, it is essential to emphasize that the chatbot was trained only until September 2021 and does not have access to the Internet. Regarding this specific aspect, a potential advantage could be seen in Google, since its links are constantly updated and accessible to patients for further reading. Its limits and advantages have already been described [14]. In our opinion, the sense is that of attempting to integrate these forms of technology, striving to improve them. For example, regarding potentially dangerous episodes of AI hallucination, ChatGPT-4 appears to be capable of recognizing this phenomenon when analyzed in a separate session [14,15]. One reflection we wish to emphasize is that these tools demonstrate various levels of competence in medical applications despite not being specifically trained for data analysis in this field.

As for pediatric orthopedics, this study yielded comparable average scores for Google Bard (3.5 ± 1) and ChatGPT (3.8 ± 0.83), with no statistically significant difference between the two platforms (*p* = 0.3092). While both chatbots offered responses of moderate to good quality, there were occasional gaps in coverage, particularly in addressing critical topics such as the timing of operating on flat feet. As reported in the results, the question that received the lowest rating was related to the timing of surgery for flat feet, to which both experts gave a Global Quality Score of 2 to 3. This low score indicates a significant gap in the chatbots’ ability to provide detailed and accurate information on this critical topic. The timing of surgical intervention for flat feet is a complex issue that requires a nuanced understanding of various factors, including the patient’s age and symptoms and the severity of the condition. The lack of adequate responses from the chatbots on these aspects could lead to confusion or incorrect decisions by patients or their parents. This highlights once again the necessity of expert oversight by an orthopedic surgeon. Moreover, according to feedback from two expert pediatric orthopedic surgeons when choosing between the two chatbots, ChatGPT proved to be more comprehensive and concise and therefore more useful to patients. Another shared consideration was the absence of incorrect information, meaning the dissemination of erroneous, misleading or false information. In conclusion, both experts concurred that the two chatbots showed promising potential as advanced education sources for patients or their parents. However, while the overall evaluation showed responses of moderate to good quality that covered some information, important topics were sometimes missing. This evaluation should reach at least a medium-high level of 4, meaning “Good quality, most important topics covered, useful to patients” [13]. This result requires ongoing development in improving these chatbots. As a result, how reliable is this information? In our view, it is essential to alert potential users to the dangers of accepting everything from the Internet as the truth, especially in the field of pediatric orthopedics. In the past, Internet usage in this specific area of orthopedics has been analyzed, highlighting that nonprofit and academic websites were the most reliable sources [16]. Patients tend to turn to search engines in their quest for medical answers and generally consider the information they find to be reliable [17,18]. Although this study may seem to yield encouraging results regarding information coming from the Internet but with the assistance of AI, it is not entirely possible to rule out the presence of inaccurate information. In our opinion, a user capable of carefully examining the content of responses should always screen the information to be provided to patients. In fact, AI has also demonstrated a potentially dangerous phenomenon, described as “AI hallucination,” which refers to an erroneous response from the chatbot that the chatbot itself does not recognize [14,15]. The danger of this phenomenon lies precisely in the particularly convincing presentation of information that could lead the user to assume it as fact, especially if the user not sufficiently knowledgeable. Another limitation to keep in mind is the request for scientific sources from these chatbots, often leading them to provide links that are found to be inaccessible. Finally, it is essential to note that ChatGPT has training data only up to September 2021. This temporal aspect could influence the generation of responses based on guidelines that may not be up to date. On the basis of the authors’ opinion, the main improvements that should be considered for AI-based NLP models to enhance their relationship with patients are reported in Table 5.

This marks the first attempt to compare the outcomes of two major AI-based chatbots in providing patient information. Both platforms were evaluated using a transparent and unbiased methodology, with careful measures taken to eliminate potential biases, such as clearing the search history and anonymizing responses from orthopedic experts. However, this paper has several limitations that must be considered to interpret the results properly. Firstly, the decision to restrict the analysis to ten questions may represent a substantial intrinsic limitation, as it only covers part of the spectrum of possible topics or issues patients may have in the field of sports medicine. In the future, a more comprehensive assessment of AI chatbots’ capabilities could be achieved by using a broader range of questions, potentially involving diverse clinical scenarios. Furthermore, as mentioned in the document, the limited training period of ChatGPT may affect its ability to provide updated and relevant information, especially considering the continuously evolving nature of medical knowledge. Another limiting aspect is the absence of an analysis based on patient feedback. Patient perceptions and needs may differ from expert evaluations, and the omission of this perspective could reduce the completeness of the paper’s conclusions regarding practical utility for individuals seeking medical information. However, we believe that a preliminary analysis by professionals is crucial in this regard.

With these considerations in mind, the paper could undoubtedly benefit from future investigations that specifically address the identified areas of concern. This would contribute to a deeper understanding of the actual validity and capacity for knowledgeable application of chatbots in delivering medical information.

## 5. Conclusions

In conclusion, despite notable limitations, AI-based chatbots hold promise as resources for patient education in orthopedics, notably in sports medicine and pediatric orthopedics. However, their role should be complementary to specialist guidance. Specialists must engage with these tools to facilitate their evolution effectively, acknowledging the inevitable integration of AI into orthopedic practice. Continuous research is essential to ensure the safe and effective incorporation of AI into patient care. Limitations include reliance on ChatGPT-3.5 and a narrow question focus. Future research could explore the impact of using more current AI models and broaden the scope of inquiry to encompass a wider range of clinical scenarios. Additionally, efforts to enhance the training data and ensure access to reliable sources could further improve the utility of AI-based chatbots in orthopedic patient education.

## Figures and Tables

**Table 1:** Global Quality Score (GQS).

Global Quality Score	
1	Poor quality, very unlikely to be of any use to patients
2	Poor quality but some information present, of very limited use to patients
3	Some information covered but important topics missing, somewhat useful to patients
4	Good quality, most important topics covered, useful to patients
5	Excellent quality, highly useful to patients

**Table 2:** Questions and answers produced by ChatGPT and Google Bard in sports medicine.

Question	ChatGPT-3.5	Score #1	Score #2	Google Bard	Score #1	Score #2
How does one recover from an anterior cruciate ligament injury?	The healing process for an anterior cruciate ligament (ACL) injury depends on the severity of the damage and the chosen treatment options. In severe cases, ACL reconstruction surgery is often necessary, during which the damaged ligament is replaced with a graft. After the surgery, it is crucial to follow a physical therapy program aimed at strengthening the surrounding muscles, restoring joint stability, and range of motion. This process can take several months. For less severe injuries or situations where surgery is not recommended, rehabilitation may be the primary path to healing. Physical therapy, including muscle strengthening exercises, is essential for improving knee function. The use of braces or knee supports may be recommended to provide support during physical activity. Pain and inflammation management is important, and the doctor may prescribe appropriate medications. Lifestyle modifications, such as avoiding high-risk activities or taking precautions during sports, may be necessary during the recovery process. Specialized guidance is essential for an accurate evaluation and a targeted treatment plan, allowing for effective healing and the prevention of chronic knee problems.	4	3	Healing from an anterior cruciate ligament (ACL) injury can take anywhere from 6 to 12 months. The initial treatment involves rest, ice application, and the use of a brace to reduce pain and swelling. Subsequently, the patient will need to start a rehabilitation program to improve knee mobility and strength. If the injury is severe, the surgeon may recommend ACL reconstruction surgery. This procedure involves replacing the damaged ligament with a graft taken from another part of the body, such as the patellar tendon or the quadriceps tendon. Post-operative recovery is longer and more demanding than non-surgical recovery. The patient will need to follow an intensive rehabilitation program to restore knee functionality. With proper treatment and hard work, most people who experience an ACL injury can return to their daily and sports activities without issues.	4	3
Can you walk when you tear the cruciate ligament?	Yes, it is possible to walk with an anterior cruciate ligament (ACL) injury, although the ability to walk depends on the severity of the injury and the presence of any associated injuries or complications. In many cases, especially with less severe or partial ACL injuries, people can walk after an initial period of pain and instability. However, it’s important to note that a complete ACL injury can make walking unsteady, especially during activities that require changes in direction or significant loads on the knee. An ACL injury compromises the stability of the knee joint, which can result in a feeling of “giving way” or insecurity while walking. Some people may need to use braces or support devices to walk more comfortably. The best course of action if you suspect an ACL injury is to consult an orthopedic doctor or a sports medicine specialist for a thorough evaluation. An accurate diagnosis and an appropriate treatment plan are essential for optimal healing and to prevent further damage to the knee joint.	5	3	A torn anterior cruciate ligament (ACL) is an injury that can cause knee pain and instability. In some cases, it is possible to walk even with a torn ACL, but walking will likely be painful and unstable. The knee may swell, and there is a risk of further injury. Typically, it is advisable to use crutches to walk after an ACL injury. This will help protect the knee and reduce pain. Over time and with rehabilitation, it is possible to improve knee mobility and strength, making walking easier and less painful. Here are some tips for walking with a torn ACL: Use crutches to unload weight from the knee. Walk slowly and cautiously. Avoid running, jumping, or making movements that put the knee at risk. Follow the instructions of the doctor and physical therapist. If walking is too painful or unstable, it is important to consult a doctor. Surgery to reconstruct the ACL may be necessary.	4	3
How to tell if the anterior cruciate ligament is torn?	The rupture of the anterior cruciate ligament (ACL) is often accompanied by clear symptoms, but an accurate diagnosis requires a medical evaluation. Here are some common signs and symptoms that may indicate a possible ACL tear: Pain: The patient may experience sudden and sharp pain in the knee, usually in the front or lateral area. The pain can vary in intensity. Swelling: The knee can swell rapidly after the injury due to the accumulation of fluid in the joint. Instability: The sensation of instability in the knee is a typical symptom. The patient may feel as if the knee “gives way” or lacks support during movement. Popping or snapping sound: The injury may be accompanied by an audible “pop” or “snap” sound during the traumatic event. Difficulty walking: The patient may have difficulty walking steadily or supporting weight on the affected knee. Loss of range of motion: The ability to fully bend or extend the knee may be compromised. Bruising: The area around the knee may develop bruising after the injury. However, these symptoms can vary from person to person, and only a doctor can confirm an ACL tear through a physical examination, joint stability tests, and sometimes imaging tests like magnetic resonance imaging (MRI). If an ACL injury is suspected, it is important to seek immediate medical evaluation, as early diagnosis can contribute to more effective treatment and a better prognosis.	4	4	The symptoms of an anterior cruciate ligament (ACL) tear include: Severe knee pain, which may be accompanied by a snapping noise or a sense of tearing. Knee swelling, typically occurring within a few hours of the injury. Difficulty in moving the knee, especially when trying to fully extend the leg or rotate the tibia relative to the femur. Knee instability, which may manifest as a feeling of unnatural movement or joint giving way. If you experience these symptoms, it is important to consult a doctor for an accurate diagnosis. To diagnose an ACL tear, the doctor will perform a physical examination and may recommend further tests, such as: X-rays, which can rule out any bone injuries. Magnetic resonance imaging (MRI), which provides a more detailed image of the knee’s soft tissues. Arthroscopy, an examination that allows the doctor to view the inside of the knee through a small incision. The treatment for an ACL tear will depend on the severity of the injury. In the case of a complete tear, the doctor may recommend surgery to reconstruct the ligament. Surgery is generally advised for athletes and individuals who need to return to high-impact activities. If the injury is partial, the doctor may recommend non-surgical treatment, which can include: Rest Ice application Ibuprofen or other non-steroidal anti-inflammatory drugs (NSAIDs) Rehabilitation Rehabilitation is essential to restore knee mobility and strength and reduce the risk of future injuries.	5	4
Where does the anterior cruciate ligament hurt?	The anterior cruciate ligament (ACL) is a ligament located inside the knee joint, so the “pain” directly associated with the ACL may not be felt in the same way as muscles or other parts of the body. However, when the ACL ruptures or is injured, the symptoms are often perceived in the surrounding area of the knee joint. The primary pain caused by an ACL injury may be felt inside or outside the knee, but it can also radiate down the leg or in the surrounding area. Patients may experience sudden and sharp pain during the injury, accompanied by swelling and a sense of instability in the knee joint. It is important to note that the perception of pain can vary from person to person and may depend on the severity of the injury. The diagnosis and management of a potential ACL injury should be entrusted to an experienced orthopedic or sports medicine doctor.	5	3	The anterior cruciate ligament (ACL) is located inside the knee, between the femur and the tibia. Its function is to prevent the tibia from moving forward relative to the femur. When the ACL ruptures, pain is typically felt inside the knee, in the front part. The pain can be intense and may be accompanied by a snapping sound or a sense of tearing. The pain can be accompanied by other symptoms such as swelling, stiffness, and knee instability. If you experience knee pain, it is important to consult a doctor for an accurate diagnosis.	4	3
What happens if you don’t have the anterior cruciate ligament surgery?”	If an anterior cruciate ligament (ACL) injury is not surgically treated, there can be some long-term consequences. However, the appropriate treatment depends on the severity of the injury and the patient’s lifestyle Joint Instability: The absence of a stable ACL can lead to repeated episodes of knee instability, with a feeling of “giving way” or “slipping” during daily activities or sports. Meniscus Damage: Untreated ACL injuries increase the risk of meniscus injuries, which are the cartilage pads inside the knee joint. These injuries can cause pain, swelling, and limited mobility. Osteoarthritis: Over time, instability and meniscus damage can lead to the early onset of osteoarthritis, a condition in which joint cartilage deteriorates, causing chronic pain and stiffness. In some cases, especially for less active patients or partial injuries, conservative treatment with physical therapy and muscle strengthening may be effective in managing an ACL injury. However, ACL reconstruction surgery is often recommended to restore joint stability and prevent long-term joint damage. The decision should be made in consultation with a specialized medical professional.	5	4	If an untreated anterior cruciate ligament (ACL) is ruptured, the knee can become unstable and painful. This can make it difficult to walk, climb stairs, and perform other daily activities. Additionally, people with an untreated ACL have a higher risk of future knee injuries, such as meniscal injuries or arthritis. In some cases, it is possible to live a normal life without having the ACL repaired. However, it is important to be aware of the risks and potential issues that can arise from this decision. Here are some of the potential issues with not repairing a ruptured ACL: Knee Instability: Without the ACL, the tibia can move forward relative to the femur, causing a sense of instability in the knee. This can make walking and performing other daily activities difficult. Knee Pain: Knee pain may be present even after an untreated ACL injury. Pain can be exacerbated by physical activity and can interfere with daily life. Future Knee Injuries: People with an untreated ACL have a higher risk of future knee injuries, such as meniscal injuries or arthritis. If you have been diagnosed with a ruptured ACL, it is important to discuss the risks and benefits of surgery with your doctor. The number of physical therapy sessions required for a ruptured ACL depends on the severity of the injury and the person’s level of activity. In general, people who undergo ACL surgery should follow a physical therapy program of at least 6 months. The physical therapy program begins with light mobility and muscle strengthening exercises and gradually progresses to more challenging exercises. People who do not undergo surgery can also benefit from physical therapy. The physical therapy program in these cases will be less intense and will focus on reducing pain and swelling and improving mobility. On average, people who undergo ACL surgery undergo about 20–30 physical therapy sessions. People who do not undergo surgery may require fewer sessions, but it is important to follow the physical therapist’s recommendations. Here are some of the main goals of physical therapy for a ruptured ACL: Reduce pain and swelling. Improve knee mobility. Strengthen the muscles around the knee. Restore knee function Physical therapy is an important part of recovering from a ruptured ACL. With appropriate treatment, most people can safely and fully return to physical activities.	4	4
How many physical therapy sessions for a torn cruciate ligament?	The number of physical therapy sessions required for the rehabilitation of an anterior cruciate ligament (ACL) depends on the severity of the injury, the individual patient’s response, and whether surgery was performed. However, a typical rehabilitation protocol following ACL reconstruction surgery involves a series of sessions that can range from 20 to 40 or more, spread out over several months. In the early stages of rehabilitation, physical therapy sessions focus on reducing swelling, restoring range of motion, and managing pain. Subsequently, the emphasis shifts to muscle strengthening exercises, balance exercises, and functional training to improve joint stability. It’s important to emphasize that ACL rehabilitation is a gradual and personalized process, and the number of sessions can vary based on the patient’s progress and the specific requirements of their recovery. The supervision of an experienced physical therapist is essential to ensure a safe and effective recovery.	4	3	The number of physical therapy sessions required for a torn anterior cruciate ligament (ACL) depends on the severity of the injury and the individual’s activity level. In general, individuals who undergo ACL surgery should follow a physical therapy program for at least 6 months. The physical therapy program begins with light mobility and muscle strengthening exercises and gradually progresses to more challenging exercises. Individuals who do not undergo surgery can also benefit from physical therapy. In these cases, the physical therapy program is less intensive and focuses on reducing pain and swelling and improving mobility. On average, individuals undergoing ACL surgery typically attend around 20–30 physical therapy sessions. Those who do not undergo surgery may require fewer sessions, but it’s important to follow the therapist’s guidance. Here are some of the main objectives of physical therapy for a torn ACL: Reduce pain and swelling. Improve knee mobility. Strengthen the muscles around the knee. Restore knee function Physical therapy is an essential part of recovering from a torn ACL. With appropriate treatment, most individuals can safely and fully return to physical activities.	5	4
How long does it take to recover from a cruciate ligament injury?	The time required to recover from an anterior cruciate ligament (ACL) injury varies significantly based on the severity of the injury, the chosen treatment, and the individual patient’s response. For partial or less severe injuries, recovery can take from a few weeks to several months with the appropriate period of physiotherapy and rehabilitation. In the case of a complete ACL injury, the healing timeline can be longer. After ACL reconstruction surgery, patients often require 6 to 12 months or more to return to normal sports activities or daily functions, although the timing can vary depending on individual circumstances. During the recovery, the supervision of an experienced physiotherapist is essential to ensure effective and safe rehabilitation. Following the treatment plan prescribed by the doctor is crucial to optimize healing and minimize the risk of further injuries or complications.	4	4	The time required to recover from an anterior cruciate ligament (ACL) injury depends on the severity of the injury and the type of treatment received. For a complete rupture of the ACL, complete healing can take from 6 to 12 months. Surgery is generally the recommended treatment for complete ACL injuries. The surgical procedure involves replacing the torn ligament with a tendon taken from another part of the body, such as the leg or the calf. After surgery, the individual will need to follow a physiotherapy program to regain knee mobility and strength. For a partial ACL injury, healing can take from 3 to 6 months. Non-surgical treatment may be sufficient for partial ACL injuries. Non-surgical treatment involves rest, ice, compression, and elevation (RICE), as well as physiotherapy. Regardless of the type of treatment received, it’s important to follow the guidance of the doctor and the physiotherapist to promote recovery.	5	3
What’s the difference between an injury and a tear of the cruciate ligament?	An injury and a tear of the anterior cruciate ligament (ACL) represent two different levels of severity in damaging the ligament itself. ACL Injury: An ACL injury indicates damage to the anterior cruciate ligament that can vary in intensity, ranging from partial strain or stretching to complete damage. In an injury, the ACL may undergo excessive stretching or tension without a complete tear. This can cause symptoms such as pain, swelling, and joint instability. ACL Tear: An ACL tear is a more severe condition in which the ligament is completely torn or damaged to the point where it can no longer perform its function of stabilizing the knee joint. This type of injury typically requires surgical ACL reconstruction to restore joint stability In both cases, it’s important to seek medical consultation for an accurate diagnosis and determine the best treatment strategy.	4	4	The anterior cruciate ligament (ACL) is an important ligament located inside the knee. Its function is to prevent the tibia from moving forward relative to the femur. An ACL injury is damage to the ligament that can range from a simple strain to a complete tear. A strain of the ACL is a minor injury that causes stretching or straining of the ligament. Symptoms of an ACL strain include pain, swelling, and stiffness. A tear of the ACL is a more severe injury that results in a cut or tear of the ligament. Symptoms of an ACL tear include intense pain, significant swelling, and knee instability. In the case of an ACL tear, it is necessary to consult a doctor for an accurate diagnosis. The doctor can perform a physical examination and may request further tests, such as an MRI, to confirm the diagnosis. The treatment for an ACL injury depends on the severity of the injury. In the case of a strain, treatment may include rest, ice, compression, elevation (RICE), and physiotherapy. In the case of a tear, treatment may involve surgery to repair or replace the ligament. Recovery from an ACL injury takes time and commitment. With appropriate treatment, most people can safely and fully return to physical activities.	4	4
When should the anterior cruciate ligament be operated on?	Surgery for an anterior cruciate ligament (ACL) injury is often recommended in specific situations, but the decision depends on the severity of the injury and the individual needs of the patient. Common indications for ACL surgery include: Chronic Instability: When a person experiences significant knee instability due to an ACL injury, making it difficult to carry out normal daily activities or sports. Multiple Injuries: When there are associated injuries such as meniscal tears or damage to other ligaments that require concurrent surgical interventions. Active Patients: For athletes or individuals who want to return to a highly active lifestyle or sports that involve the knee, as surgery can help restore the necessary stability for such activities. Young Patients: In some cases, especially in young individuals, surgery is recommended to prevent progressive joint damage and the early onset of osteoarthritis. The decision on surgery should be made after a comprehensive evaluation by a specialized physician, taking into account the symptoms, the severity of the injury, and the patient’s lifestyle. ACL reconstruction surgery carries risks and requires a period of rehabilitation, so it should be carefully weighed against the expected benefits.	5	5	The decision to undergo anterior cruciate ligament (ACL) surgery is a complex one that should be made on a case-by-case basis. There are several factors to consider, including the person’s age, level of physical activity, and the severity of the injury. In general, surgery is recommended for young and active individuals who wish to return to high-impact sports like soccer, basketball, or skiing. Surgery can help restore knee stability and reduce the risk of future injuries. For less active individuals or those who do not wish to return to high-impact sports, non-surgical treatment may be an option. Non-surgical treatment can include rest, ice, compression, elevation (RICE), physiotherapy, and bracing. Ultimately, the decision to undergo ACL surgery is a personal one and should be based on individual needs and preferences. Here are some of the factors that can influence the decision to undergo ACL surgery: Age: Younger adults are more likely to return to high-impact sports after ACL surgery. Level of Physical Activity: People who are active and engage in high-impact sports are more likely to benefit from ACL surgery. Severity of the Injury: Complete ACL tears are more likely to require surgery than partial tears. Presence of Other Injuries: The presence of other injuries, such as meniscal tears or collateral ligament injuries, can influence the decision to undergo ACL surgery. It’s important to discuss all of these factors with your doctor to make the best decision for your health.	4	5
How long to wait before undergoing ACL surgery	The ideal timing for anterior cruciate ligament (ACL) reconstruction surgery can vary based on the individual circumstances of the patient and the recommendations of the orthopedic surgeon. However, there are some general guidelines to consider: Swelling and Inflammation Control: Before surgery, it is important for the knee to have reached a relatively stable condition with swelling and inflammation under control. This may take several weeks to several months after the initial injury. Range of Motion Restoration: It is necessary to restore as much knee range of motion as possible before surgery. Physical therapy can be used to improve joint flexibility. Muscle Strength Control: Strengthening the muscles surrounding the knee is crucial before surgery, as well-developed muscles can contribute to a better post-operative recovery. Discussion with the Doctor: The decision of when to have surgery should be made in collaboration with the orthopedic surgeon, taking into account the patient’s symptoms, joint stability, and other individual factors. In general, ACL reconstruction surgery is performed once the knee is stable, swelling is under control, and good physical preparation has been achieved. However, the timing can vary from case to case, so it is essential to follow the doctor’s recommendations.	5	4	The waiting time before undergoing ACL surgery is a decision that must be made on a case-by-case basis, considering the severity of the injury, the person’s age, and their activity level. In general, it is advisable to perform ACL surgery as soon as possible after the injury to reduce the risk of further injuries and improve the chances of recovery. However, in some cases, it may be necessary to wait before surgery. For example, if the injury is recent and the knee is still swollen, the doctor may recommend waiting for a few weeks before surgery. This is because surgery can be more difficult and painful if the knee is swollen. In other cases, the doctor may recommend waiting until the person has completed non-surgical treatment, such as rest, ice, compression, elevation (RICE), and physiotherapy. This is because non-surgical treatment can help reduce pain and swelling and improve knee mobility. Ultimately, the decision of when to undergo ACL surgery is a personal one that should be made based on individual needs and preferences. It is important to discuss all the factors involved with your doctor to make the best decision for your health. Here are some of the factors that can influence the decision of when to undergo ACL surgery: Severity of the Injury: Complete ACL injuries typically require more urgent surgery compared to partial injuries. Age of the Person: Younger adults are more likely to fully recover from ACL surgery compared to older adults. Level of Physical Activity: Individuals who are active and engage in high-impact sports are more likely to benefit from earlier ACL surgery. Presence of Other Injuries: The presence of other injuries, such as meniscal tears or collateral ligament injuries, can influence the timing of ACL surgery.	4	5

**Table 3:** Questions and answers produced by ChatGPT and Google Bard in pediatric orthopedics.

Question	ChatGPT-3.5	Score #1	Score #2	Google Bard	Score #1	Score #2
What problems does flat feet pose?	Flat foot, or pes valgus, is a condition in which the natural arch of the foot is reduced or absent, causing a flattening of the foot when it makes contact with the ground. This condition can lead to various problems and discomfort: Pain: Flat feet can cause pain in the feet, ankles, knees, hips, and even the back, as the lack of support can put additional pressure on these joints. Fatigue: People with flat feet may experience early fatigue when walking or standing for extended periods. Instability: The lack of a proper arch can make the lower limb less stable, increasing the risk of injuries. Footwear issues: Finding comfortable and appropriate shoes can become challenging. Deformities: In some cases, flat feet can lead to deformities such as hallux valgus (bunions). The treatment of flat feet depends on the severity of symptoms and may include strengthening exercises, custom orthotics, physical therapy, or, in some cases, surgery. to correct the arch. Early management is important to prevent long-term issues	5	5	Flat feet is a condition in which the arch of the foot, which is the natural curvature of the foot, is absent or reduced. This can lead to a range of problems, including: Pain: Flat feet can cause pain in the foot, ankle, knee, and back. Muscular fatigue: Flat feet can fatigue the muscles of the foot, ankle, and leg. Balance issues: Flat feet can make it difficult to maintain balance, especially when walking or running. Knee instability: Flat feet can increase the risk of knee injuries, such as sprains and tears. Joint problems: Flat feet can contribute to osteoarthritis of the foot, ankle, and knee. Flat feet can be congenital, present from birth, or acquired, developing over the course of one’s life. Congenital flat feet are often caused by genetic issues or developmental defects. Acquired flat feet can be caused by various factors, including: Trauma: Foot injuries, such as fractures, can damage the muscles and ligaments of the foot and contribute to the development of flat feet. Neurological diseases: Neurological diseases, such as spina bifida, can damage the muscles and ligaments of the foot and contribute to flat foot development. Metabolic diseases: Metabolic diseases, like diabetes, can damage the nerves and muscles of the foot and contribute to flat feet. The treatment for flat feet depends on the cause and severity of the condition. In some cases, flat feet can be managed with exercises and physical therapy. In other cases, orthopedic inserts may be necessary, or in more severe cases, surgery may be required. Here are some tips to prevent flat feet: Wear comfortable and well-supported shoes. Do foot and ankle muscle strengthening exercises. Maintain a healthy weight.	3	3
How is flat feet treated?	The treatment for flat feet depends on the severity of symptoms and the underlying cause. Here are some treatment options: Muscle strengthening exercises: Targeted exercises to strengthen the muscles of the foot, ankle, and leg can help improve stability and arch support. Orthopedic inserts: The use of custom-made or over-the-counter orthotic inserts can provide additional arch support and improve posture. Physical therapy: Physical therapy may be prescribed to improve mobility, muscle strength, and balance. Lifestyle changes: Avoiding inappropriate footwear and adopting proper posture can contribute to the treatment. Surgery: In severe cases or when flat feet are caused by a structural deformity, surgery may be necessary to reconfigure the arch and correct the issue. It’s important to consult with an orthopedic specialist or podiatrist for an accurate evaluation and appropriate treatment planning. Early management can help prevent long-term complications and improve the quality of life for those with flat feet.	4	4	The treatment for flat feet depends on the cause and severity of the condition. In some cases, flat feet can be managed with exercises and physical therapy. In other cases, orthotic inserts may be necessary, or in more severe cases, surgery may be required. Exercises and Physical Therapy Exercises and physical therapy can help strengthen the muscles of the foot and ankle, which can provide support for the arch. Some common exercises include Stretching exercises: Stretching exercises can improve the flexibility of the foot and ankle muscles. Strengthening exercises: Strengthening exercises can help build strength in the foot and ankle muscles. Balance exercises: Balance exercises can improve balance and coordination. Orthotic Inserts Orthotic inserts are custom-made devices that provide support to the arch. They can be helpful in relieving pain and improving foot function. Surgery Surgery is a rare measure but may be necessary in some cases, such as severe flat feet or persistent pain that doesn’t respond to other treatments. Surgery can be used to correct foot deformities and restore the arch. Home Care There are some things you can do at home to help manage flat feet, such as: Wear comfortable and well-supported shoes. Avoid activities that cause pain. Apply ice or warm compresses to alleviate pain. Take pain-relieving medications if necessary. It’s important to consult a doctor to determine the best treatment for flat feet.	3	5
How can you tell if you have flat feet?	To determine if you have flat feet, you can make some simple observations and perform tests at home: Arch of the Foot: By examining your foot from a side view, you can check if there is a natural arch in the central area of the foot. In a normal foot, there should be an upward curve. Wet Footprint Test: Wet the soles of your feet and walk on a surface like a tiled floor. The footprint left behind can help identify flat feet, as the arch will be missing. Symptoms: Foot pain, ankle pain, knee pain, or back pain, early fatigue during walking, or a feeling of instability can be indicators of flat feet. However, for an accurate diagnosis and treatment planning, it’s important to consult a healthcare professional, such as an orthopedic specialist or a podiatrist. They can conduct a comprehensive evaluation, including arch measurement, and recommend appropriate treatment options if needed	3	4	Flat feet are a condition in which the arch of the foot, which is the natural curvature of the foot, is absent or reduced. This can lead to a range of problems, including pain, muscle fatigue, balance issues, and knee instability. Signs and symptoms of flat feet may include: Feet appearing flat when standing. Pain in the feet, ankles, knees, or back. Muscle fatigue in the feet, ankles, or legs. Balance problems, especially when walking or running. Knee instability, especially when walking or running. Joint issues, such as osteoarthritis. If one or more of these symptoms are present, it’s important to consult a doctor for a flat feet diagnosis. The doctor can perform a physical examination to assess the foot’s condition. They may also request X-rays or other imaging tests to rule out other conditions. If flat feet are diagnosed, the doctor can recommend appropriate treatment, which may include exercises, physical therapy, orthotic inserts, or, in more severe cases, surgery. Here are some tips to prevent flat feet: Wear comfortable and well-supported shoes. Perform foot and ankle muscle strengthening exercises. Maintain a healthy weight.	2	4
Which sports are suitable for flat feet?	The choice of sports for individuals with flat feet should take into account the severity of the condition and recommendations from a doctor or physiotherapist. In general, many people with flat feet can participate in a variety of sports, but they may need to consider the following: Low-Impact Sports: Activities such as swimming, cycling, yoga, and Pilates are often well-tolerated by people with flat feet as they reduce the impact on the feet and joints. Strength-Building Exercises: Muscle strengthening exercises, such as physical therapy, can be helpful in improving stability and preventing injuries during physical activities. Appropriate Footwear: Regardless of the chosen sport, it’s essential to use suitable footwear and, in some cases, orthotic inserts to provide the necessary support. Avoiding High-Impact Sports: Sports like long-distance running or basketball can put excessive pressure on the feet and joints and may not be the best choice for individuals with flat feet. In any case, consult a healthcare professional to get specific advice on sports participation based on your individual condition.	5	3	People with flat feet can engage in a variety of sports. However, it’s important to choose sports that do not strain the feet and joints. Some sports suitable for individuals with flat feet include: Swimming: Swimming is an excellent sport for people with flat feet, as it does not strain the feet. Cycling: Cycling is another low-impact sport suitable for people with flat feet. Hiking: Hiking is a great way to stay active without overloading the feet. Yoga: Yoga is an excellent way to strengthen the muscles of the foot and ankle. Pilates: Pilates is another great way to strengthen the muscles of the foot and ankle. Other sports that can be practiced by people with flat feet but require more support include: Soccer: People with flat feet can play soccer, but it’s important to wear shoes with good arch support. Basketball: People with flat feet can play basketball, but it’s important to wear shoes with good arch support and engage in regular stretching. Running: People with flat feet can run, but it’s important to start gradually and wear shoes with good arch support. It’s important to consult a doctor or orthopedic specialist before starting a new sport, especially if you have flat feet.	3	4
What happens if flat feet are not treated?	If not treated properly, flat feet can lead to a range of long-term problems. These consequences may include: Chronic Pain: Flat feet can cause persistent pain in the feet, ankles, knees, and back due to the lack of support and alteration of walking biomechanics. Structural Deformities: In some individuals, flat feet can lead to structural deformities such as bunions (hallux valgus) or hammertoes. Increased Risk of Injuries: Inadequate support increases the risk of muscle and joint injuries, especially during physical activities. Mobility Difficulties: Flat feet can limit the ability to walk and perform daily activities. Postural Problems: It can affect overall body posture and lead to balance issues. It’s essential to seek medical consultation if you suspect you have flat feet to start appropriate treatment and prevent these complications.	4	3	If flat feet are not treated, a range of problems can occur, including: Pain: Flat feet can cause pain in the feet, ankles, knees, and back. Muscle Fatigue: Flat feet can fatigue the muscles in the feet, ankles, and legs. Balance Issues: Flat feet can make it difficult to maintain balance, especially when walking or running. Knee Instability: Flat feet can increase the risk of knee injuries, such as sprains and tears. Joint Problems: Flat feet can contribute to arthritis in the feet, ankles, and knees. In severe cases, flat feet can lead to walking difficulties and gait issues. If you have flat feet, it’s important to consult a doctor to determine the best treatment. Treatment may include exercises, physical therapy, orthotic inserts, or, in more severe cases, surgery.	3	3
What causes flat feet?	Flat feet can have various causes, and it’s important to identify the specific cause for appropriate treatment. Some of the main causes of flat feet include: Congenital Flat Feet: Some individuals are born with less pronounced arches due to genetic predisposition. Acquired Flat Feet: Flat feet can develop during one’s lifetime due to factors such as aging, obesity, wear and tear on the tendons supporting the arch, or foot injuries. Injuries: An injury to the tendons or joints of the foot can lead to a reduction in the arch and the development of flat feet. Neuromuscular Conditions: Certain conditions like cerebral palsy or damage to the sciatic nerve can affect foot biomechanics and result in flat feet. Developmental Issues: In some cases, flat feet may arise from developmental problems in the bones or joints of the foot during childhood. An evaluation by a healthcare professional is essential to determine the specific cause and plan the most appropriate treatment	4	4	Flat feet are a condition in which the natural arch of the foot is absent or reduced. This can be attributed to various factors, including: Genetics: Flat feet can be a genetic condition passed from parents to children. Injuries: Foot injuries, such as fractures, can damage the muscles and ligaments of the foot and contribute to the development of flat feet. Neurological Disorders: Neurological conditions like spina bifida can harm the muscles and ligaments of the foot and play a role in the development of flat feet. Metabolic Diseases: Metabolic disorders, such as diabetes, can damage the nerves and muscles of the foot, contributing to flat feet. Obesity: Obesity can increase the risk of developing flat feet. Flat feet can be congenital, present from birth, or acquired, developing over one’s lifetime. Congenital flat feet are often caused by genetic issues or developmental defects. Acquired flat feet can result from various factors, as mentioned above. In general, flat feet are a benign condition that doesn’t typically lead to serious problems. However, in some cases, flat feet can cause pain and other issues, as listed above. In such instances, treatment may be necessary, including exercises, physical therapy, orthopedic insoles, or, in more severe cases, surgery.	3	3
When to operate flat feet?	Surgery for flat feet is typically considered only when conservative treatments, such as orthopedic insoles, physical therapy, and muscle strengthening exercises, have failed to improve symptoms or when the severity of flat feet results in significant limitations in the quality of life or significant structural deformity. Surgical indications may include: Persistent pain: If foot or joint pain continues despite conservative treatment. Structural deformity: If a structural deformity like bunions (hallux valgus) or hammer toes develops as a result of flat feet. Mobility difficulties: When flat feet significantly limit the ability to walk or participate in daily activities. The decision for surgery should be made in collaboration with a specialized orthopedic surgeon, taking into account individual symptoms, the severity of the condition, and the patient’s expectations.	3	2	Surgery for flat feet is a rare measure but may be necessary in some cases, such as: Severe flat feet: Severe flat feet can cause pain and other problems, as listed above. Persistent pain: Persistent pain that doesn’t respond to other treatments like exercises, physical therapy, or orthopedic insoles. Walking difficulties: Walking problems that interfere with daily life. Neurological or metabolic conditions: Flat feet can be a symptom of other conditions like spina bifida or diabetes. In these cases, surgery may be necessary to correct the underlying condition. Surgery for flat feet can be performed in various ways, depending on the cause and severity of the condition. The most common types of surgery include: Tendon transfer: This involves transferring a tendon to provide better arch support. Osteotomy: The surgeon cuts and repositions one or more bones in the foot to correct deformities. Fusion: Fusion surgery involves fusing two or more bones in the foot to stabilize the arch. Surgery for flat feet is generally safe and effective. However, like any surgical procedure, it carries risks, including: Infection Bleeding Nerve or blood vessel damage Anesthesia risks After surgery, it is important to follow the doctor’s instructions for proper healing.	2	2
How to treat adult flat feet?	To address flat feet in adults, you can consider the following treatment options: Orthopedic insoles: Using custom-made or over-the-counter orthotic insoles can provide support for the arch of the foot and improve posture. It’s important to consult with an orthopedist or a podiatrist to get insoles tailored to individual needs. Strengthening exercises: Performing specific exercises to strengthen the muscles in the legs and feet can help improve stability and arch mobility. Physical therapy: A physical therapist can develop a personalized treatment program to enhance muscle strength, flexibility, and balance. Proper footwear: Wearing appropriate shoes with good arch support is crucial in reducing symptoms. Weight loss: If obesity contributes to flat feet, weight loss can help alleviate symptoms. Surgery: Surgery is a rare option but may be considered when symptoms are severe and do not respond to other therapies. It’s essential to consult with a healthcare professional for a comprehensive evaluation and a treatment plan tailored to your individual situation.	4	3	Adult flat feet can be treated in various ways, depending on the severity of the condition. For mild flat feet, treatment may include: Comfortable and well-supported shoes: Shoes with good arch support can help reduce pain and inflammation. Exercises and physical therapy: Exercises and physical therapy can help strengthen the foot and ankle muscles, which can support the arch. For severe flat feet, treatment may include: Orthopedic insoles: Custom-made orthotic insoles provide arch support and can be helpful in alleviating pain and improving foot function. Surgery: Surgery is a rare measure but may be necessary in some cases, such as severe flat feet or persistent pain that doesn’t respond to other treatments. Here are some tips for adults with flat feet: Wear comfortable and well-supported shoes: Properly fitting shoes with good arch support are essential for people with flat feet. Engage in exercises and physical therapy: These can help strengthen the foot and ankle muscles, which support the arch. Maintain a healthy weight: Obesity can increase the risk of developing flat feet and worsening symptoms. Avoid activities that cause pain: It’s important to avoid activities that cause foot pain. Take pain-relieving medication if necessary: Pain relievers can be helpful for acute pain. If you experience one or more flat feet symptoms, it’s important to consult a doctor for a proper diagnosis and appropriate treatment.	5	4
How do you walk after flat foot surgery?	After surgery to correct flat feet, the walking pattern will gradually change during the rehabilitation period. Here’s an overview of what the post-operative walking process might look like: Immediately after surgery: Initially, the patient may need to use crutches or a brace to avoid direct weight-bearing on the operated foot. The foot may be wrapped or placed in a special brace. Rehabilitation: Over time, the patient will begin physical therapy to regain strength and mobility. Specific exercises will help strengthen the leg and foot muscles. Gradual weight-bearing: Gradually, the patient will start to put weight on the operated foot. This process will be guided by the physical therapist or orthopedic surgeon. Full recovery: Achieving a complete return to a normal gait may take several weeks or months. The patient will need to carefully follow the instructions of the doctor and physical therapist to achieve a safe and pain-free walking pattern. The duration of the process varies depending on the nature of the surgery and the individual patient’s response to rehabilitation.	4	3	The way you walk after flat foot surgery can vary depending on the type of surgery performed and the severity of the condition. In general, it’s essential to follow the doctor’s instructions for a proper recovery. After surgery, the foot is likely to be swollen and painful. The doctor may prescribe pain medications to reduce pain and inflammation. Initially, you may need to wear a brace or cast to protect the foot during the healing process. The doctor will indicate when it’s possible to start walking without the brace or cast. Walking can be challenging and painful at first. It’s important to start slowly and gradually increase the time and distance you walk. Here are some tips for walking after flat foot surgery: Start gradually: Don’t try to walk too long or too fast initially. Use a cane or walker: A cane or walker can help support your weight and reduce pain. Walk on a flat surface: Avoid walking on uneven surfaces, such as stairs or uneven sidewalks. Take frequent breaks: Stop and rest if you experience pain. If you experience one or more of the following symptoms, it’s essential to consult the doctor: Severe pain Excessive swelling Discoloration Fever With proper recovery, most people can walk normally after flat foot surgery.	4	4
How to alleviate flat foot pain?	To alleviate the pain associated with flat feet, you can take a series of measures and treatments: Orthopedic insoles: Using custom-made or over-the-counter orthotic insoles can provide the necessary arch support and reduce pain. Proper footwear: Choosing shoes with arch support and adequate cushioning can alleviate discomfort. Strengthening exercises: Performing specific exercises to strengthen leg and foot muscles can help improve stability and reduce pain. Physical therapy: A physical therapist can develop a personalized treatment program to enhance mobility and muscle strength. Anti-inflammatories: In some cases, the use of non-steroidal anti-inflammatory drugs (NSAIDs) can help reduce pain and inflammation. Rest: Reducing physical activity and giving the foot time to recover may be necessary during periods of acute pain. Medical consultation: Consulting with an orthopedist or podiatrist for a comprehensive evaluation and appropriate treatment plan is essential for managing flat foot pain.	5	4	There are several ways to alleviate flat foot pain. Here are some tips: Wear comfortable and well-supported shoes: Shoes with good arch support can help reduce pain and inflammation. The shoes should be the right size and should not be too tight. Engage in exercises and physical therapy: Exercises and physical therapy can help strengthen the muscles of the foot and ankle, which can support the arch. Maintain a healthy weight: Obesity can increase the risk of developing flat feet and worsening symptoms. Avoid activities that cause pain: It’s important to avoid activities that cause foot pain. Take pain relievers, if necessary: Pain relievers can be helpful for acute pain. Here are some specific exercises that can help alleviate flat foot pain: Exercise 1: Raise the heel off the ground and hold it up for 5 s, then lower it. Repeat 10 times. Exercise 2: Bend the toes upward and then downward. Repeat 10 times. Exercise 3: Lift the heel off the ground and rotate the foot clockwise and counterclockwise. Repeat 10 times in each direction. If the pain is severe or doesn’t respond to other treatments, your doctor may recommend the use of orthopedic insoles or surgery.	5	5

**Table 4:** Average scores of Google Bard and ChatGPT.

Sports Medicine	
Google Bard	Average score 4 ± 0.78
ChatGPT	Average score 4.1 ± 0.7
No statistically significant difference	(*p* = 0.6787)
**Pediatric Orthopedics**	
Google Bard	Average score 3.5 ± 1
ChatGPT	Average score 3.8 ± 0.83
No statistically significant difference	(*p* = 0.3092)

**Table 5:** Potential AI-based NLP model improvements.

Source Accessibility	Platforms should ensure access to reliable and up-to-date sources for providing medical information. This could involve improving the management of hyperlinks, ensuring that they are always accessible and current.
Quality Control of Responses	It is essential to implement systems to ensure the accuracy and reliability of the information provided. This could include automatic verification of responses before publication and the ability for users to report incorrect or misleading content.
Continuous Updates	Given the rapid advancements in the field of medicine, platforms should be continuously updated with the latest discoveries and clinical guidelines. This may require frequent review and integration of training data to ensure the relevance and accuracy of the information provided.
Patient Engagement	Integrating patient feedback into the development and improvement process of AI platforms can help identify areas for improvement and personalize the user experience to meet individual needs.
Transparency and Accountability	Platforms should be transparent about their capabilities and limitations, educating users on the proper use of the information provided and encouraging critical evaluation of content. Additionally, they should be clear about the data used for training and the sources of the information provided.

## Data Availability

The data presented in this study are available within the manuscript.
